# Cognitive performance of individuals with animal hoarding

**DOI:** 10.1186/s12955-020-01288-1

**Published:** 2020-02-24

**Authors:** Luis Henrique Paloski, Elisa Arrienti Ferreira, Dalton Breno Costa, Camila Rosa de Oliveira, Carmen Moret-Tatay, Tatiana Quarti Irigaray

**Affiliations:** 1 Pontifícia Universidade Católica do Rio Grande do Sul – PUCRS, Av. Ipiranga, 6681, Building 11, 9th floor, Porto Alegre, RS CEP 90619-900 Brazil; 2 Faculdade Meridional – IMED, Rua Senador Pinheiro, 304, Passo Fundo, RS CEP 99070-220 Brazil; 3 Universidade Federal de Ciências da Saúde de Porto Alegre – UFCSPA, Rua Sarmento Leite, 245, Porto Alegre, RS CEP 90050170 Brazil; 4 Facultad de Psicología, Universidad Católica de Valencia San Vicente Mártir, Valencia, Spain

**Keywords:** Animal hoarding, Cognitive profile, Executive functions

## Abstract

**Background:**

The purpose of this study was to characterize the cognitive performance of individuals with animal hoarding.

**Methods:**

This is a cross-sectional study, in which 33 individuals between the ages of 29 to 84 (*M* = 61.39; *SD* = 12.69) with animal hoarding have been assessed. The participants completed a neurocognitive battery including measures of general cognitive functioning, visual memory and organization, verbal fluency, and verbal reasoning.

**Results:**

Data suggest that individuals with animal hoarding have high rates of cognitive deficits related to visual memory and verbal reasoning.

**Conclusions:**

Based on the performance tests used, we can suggest the existence of cognitive difficulties related especially to the executive functions of individuals with animal hoarding in this sample.

## Introduction

Animal hoarding is considered a new field of research and there are few empiric studies on the topic. It is defined by the accumulation of a large number of animals and a failure to provide minimal standards of nutrition, sanitation and veterinary care (American Psychiatric Association 2014). Additionally, there is the subject’s inability to act on the deteriorating conditions of the animals (starvation, disease, death) and a denial or downplaying of the hoarding-derived issues both for the animals and people sharing the same space [7, 8, 12].

In DSM-5, the animal hoarding is treated as a special manifestation of the Hoarding Disorder and some studies discuss these differences [10, 35]. It is different mostly given the animal hoarding’ poorer insight [1]. The animal hoarding differs from the object hoarding from its nature, because animals require more interaction and attention than objects. Also, the majority of individuals who hoards animals do not present the behavior of hoarding objects [9].

Individuals who accumulate animals may have their cognitive abilities compromised [4, 19, 37]. Likewise, it is believed that animal hoarding may stem from cognitive deficits, possibly more severe ones, given the unhealthier environment in which individuals and animals live (American Psychiatric Association 2014).

Studies suggest that attention deficit disorders are found in hoarding disorders, and it is estimated that 28% of individuals fulfill the criteria for the Attention Deficit Disorder/Hyperactivity subtype [6, 10, 11]. Other studies suggest low levels of alternating attention [14] and selective attention [19] are related to hoarding disorders.

In addition to attention, other cognitive functions are compromised in hoarding disorders, such as memory [12, 15], and so are executive functions [36]. Information processing and categorization and decision-making skills are also compromised [33].

Blom et al. [4] found a relationship between hoarding disorders and deficits in memory and decision-making skills. Moreover, deficits in terms of categorization, information processing speeds, and verbal memory were found as well [19]. Moshier et al., [22] point out that, although studies show perform below standard in neuropsychological tests in terms of working memory, focused attention, and executive performance tasks, such information is still insufficient for building a proper profile of individuals’ cognitive performance.

Although this paper is on animal hoarding, no empiric studies looking into the cognitive performance of people with this characteristic were found. It is important to investigate cognitive abilities of animal hoarding in order to be possible to develop specific therapeutic interventions for this population [25]. Therefore, our literature review comprised studies on Hoarding Disorder. The main purpose of this study is to investigate the cognitive performance of animal hoarding. The core hypothesis of this study is that, given there are cognitive performance deficits in object accumulation, we infer cognitive deficits may be found in animal hoarding [13, 15, 21, 27].

## Method

### Participants

Seventy-five probable cases of animal hoarding were found from administrative proceedings filed in the city of Porto Alegre by the City Office for Animal Rights (SEDA, in the Portuguese acronym). The researchers visited 61 homes between August 2015 and May 2016. The team was allowed in by 48 people, 38 of whom accepted to take part in the study. The addresses to the other 14 homes were either not found by the team or the people had already moved.

We used the DSM-5 (American Psychiatric Association 2014) diagnostic criteria to define the people with Animal Hoarding. They are: (1) the accumulation of a large number of animals; (2) a failure to provide minimal standards of nutrition, sanitation, and veterinary care; (3) a failure to act on the deteriorating condition of the animals (eg, disease, starvation, death) and the environment (eg, severe overcrowding, extremely unsanitary conditions).

Out of the 38 people who agreed to participate, three did not meet the criteria required to be diagnosed with Animal Hoarding Disorder because their animals were in good health and nutrition conditions and the environment was suitable. Regarding this criterion, a report on each home visited and provided by SEDA veterinarians was used. One participant was excluded because of a speech impediment that prevented data from being collected. Another one was also excluded for being a schizophrenic.

### Instruments

#### Socio-demographic data form

Comprised the age, gender, marital status, schooling, and income variables. We also investigated the number and species of animals in the home, as well as the time when hoarding began.

#### Mini-mental state examination (MMSE)

The MMSE is a general cognitive screen. It contains questions that evaluate time and space orientation, registration of three words, attention and calculation, recall of three words, language, and visuoconstructional ability. The Portuguese version translated by [3] was used. We used the cutoff point suggested in the study by Kochhann et al. [17] to characterize the participants with and without a deficit according to their schooling: 21 for the group of illiterates, 22 in the low schooling group (1–5 years of education), 23 in the mid-schooling group (6–11 years of education), and 24 in the highly educated group (≥12 years of education). These values are based on the study by Kochhann et al. [17] among elderly people in southern Brazil. The study by Santos et al. [29] found a 0.80 Cronbach’s alpha.

#### Rey complex figures

They are complex, geometric, abstract figures made of various parts. Figures must be copied and after 3 minutes drawn from memory [23, 24]. This test evaluates perception, visuoconstructional ability, and visual memory. Additionally, it makes it possible to evaluate planning, organization, problem-solving strategies, and motor function skills [26]. The Rey complex figure test shows good internal consistency, according to the Cronbach’s alpha coefficient estimated at 0.86 when copying and 0.81 when drawing from memory [23].

#### Semantic verbal fluency test - category animals

In this test, participants are asked to produce as many animal species as possible over 1 minute. It is a measurement of executive functions which primarily evaluates verbal fluency. It also measures a person’s ability to organize their thoughts and the strategies they use to find words. A participant’s score is the total sum of animals listed in 1 minute, minus repeated words [32]. In the study by Santos [30], Cronbach’s alpha was 0.74.

#### WASI - Wechsler abbreviated scale of intelligence similarities subtest

WASI is a short test to measure intelligence and typically lasts 30 to 45 min on average. This study used only the Similarities subtest, whose main purpose is to measure verbal concept formation, abstract verbal reasoning, and general intellectual skills. The subtest shows good internal consistency, ranging from 0.84 to 0.96 [34].

### Data collection procedures

The project was approved by the PUCRS Ethics Committee (CEP-PUCRS) under CAAE: 44489715.8.0000.5336. The participants were contacted via home visits, and those who accepted to take part in the study signed an informed consent form. Next, they individually answered the questionnaires. Participants were evaluated during home visits lasting 1 hour and a half, on average, by the project coordinator and team. The latter comprised psychologists and Psychology research undergrads trained beforehand to help administer the assessments used in this study.

The city of Porto Alegre, via SEDA, provided a list of 75 hoarding cases and their contact information, as well as a veterinary doctor and an inspector to accompany the researchers during their visits to participants. While the team evaluated the participants, the animals were assessed and treated by the veterinarians. In case an animal required some sort of specialized treatment, it was taken to the animal hospital upon the participants’ permission. The Environmental Division at the Rio Grande do Sul State Attorney’s Office (MPRS) provided transportation for PUCRS faculty and students upon prior scheduling. The interviews with the participants were conducted in the vehicle provided by the MPRS. The researchers took the necessary care to make the vehicle appropriate for the instruments application.

### Data analysis procedures

The assessments were administered and graded according to their respective manuals or rules. The tests containing normative data for the Brazilian population were analyzed in terms of percentiles and T scores. The T scores were used only to identify whether the participant’s performance was deficient or not. The T score is a standardized score and is verified by normative data.

The total Rey complex figure test scores were converted into percentiles according to the manual [24]. Then, they were reclassified into adequate and substandard performance, that is, percentiles from 50 to 100 were reclassified as adequate and from 10 to 40 as substandard. Gross verbal fluency test scores were converted into Z scores according to the participants’ schooling [5], where Z ≥ − 1.3 scores were considered substandard performance [18]. The Similarities subtest was graded according to its manual [34]. First, gross scores were calculated. Then, they were converted into T scores according to the participants’ age. Next, T scores were reclassified as follows: up to 50 adequate and < 50 as substandard.

The information was organized and analyzed using a bank created through the Statistical Package for the Social Sciences (SPSS, version 17) software for Windows. The information was described by means of absolute (*n*) and relative (%) frequencies for qualitative variables, and by mean and standard deviation for quantitative variables. In order to present more detailed cognitive characteristics, participants were divided into two age groups (20–59 years and 60+ years), corresponding to young and older adults.

## Results

Table 1 shows sociodemographic and animal hoarding data of each participant.

**Table 1:** Sociodemographic and animal hoarding data of each participant

Participant	Age (year)	Education (year)	Sex	Type of animal	Number of animals
1	58	11	Female	Dogs and cats	56
2	72	11	Female	Dogs and cats	101
3	62	10	Female	Dogs	80
4	83	15	Male	Dogs and cats	71
5	44	7	Female	Dogs and cats	32
6	77	1	Female	Dogs	12
7	76	7	Female	Dogs and cats	34
8	69	9	Female	Dogs and cats	41
9	53	8	Male	Dogs and cats	34
10	61	8	Male	Dogs	40
11	67	3	Male	Dogs	40
12	65	13	Female	Dogs and cats	3
13	49	16	Female	Dogs and cats	62
14	53	11	Female	Dogs and cats	59
15	78	11	Female	Dogs and cats	73
16	62	9	Female	Dogs and cats	30
17	36	11	Female	Cats	25
18	73	16	Female	Cats	20
19	38	4	Male	Dogs and cats	16
20	29	16	Male	Dogs and cats	49
21	64	3	Female	Dogs	60
22	61	11	Female	Dogs	6
23	56	8	Female	Dogs	65
24	58	12	Male	Dogs	19
25	60	11	Female	Dogs and cats	33
26	71	4	Female	Dogs	20
27	55	11	Male	Dogs and ducks	75
28	56	16	Female	Dogs and cats	26
29	63	10	Female	Dogs and cats	71
30	63	7	Female	Dogs and cats	28
31	64	5	Male	Dogs	11
32	66	15	Female	Cats	47
33	84	0	Female	Dogs	18

Table 2 shows the description of socio-demographic profiles, incidence of psychopathological symptoms, and cognitive performance of adults with animal hoarding. The final sample comprised 33 people ranging in age between 29 and 84 years (*M* = 61.39; *SD* = 12.69), 64% of whom were over 60 years old. Of the participants, 73% were female and 27% male, with schooling between one and 16 years (*M* = 9.39; *SD* = 4.40). Income-wise, 75% earned between one and two minimum monthly salaries. It was found that 90% of participants were single and 51% lived on their own. The number of animals per home ranged between 3 to 101 (*M* = 41.12; *SD* = 24.41), totaling 1357 animals, and the time in years that hoards animals ranged from 3 to 70 years (*M* = 23.09; *SD* = 15.98). No significant associations were found between number of animals and education (*r* = .273, *p* = .124), age (*r* = .052, *p* = .774), sex (Х^2^ = .248, *p* = .619) and income (Х^2^ = .010, *p* = .922). Also, the time that hoards animals did not present significant associations with schooling (*r* = − 081, *p* = .653), sex (Х^2^ = .508, *p* = .476), and income (Х^2^ = .272, *p* = .602). However, there was a positive and moderate association between age and time that hoarded animals (*r* = .393, *p* = .024).

**Table 2:** Sociodemographic and cognitive characteristics of participants

	*n*	%	*M*	*SD*	Minimum - maximum
Gender					
Female	24	36,40	–	–	–
Male	9	63,60	–	–	–
Marital status					
Single	22	66,67	–	–	–
Married	4	12,12	–	–	–
Separate	3	9,09	–	–	–
Widower	4	12,12	–	–	–
Income^a^					
1 a 2 MS	25	75,76	–	–	–
3 a 4 MS	3	9,09	–	–	–
5 a 6 MS	2	6,06	–	–	–
7 a 8 MS	1	3,03	–	–	–
More than 10 MS	2	6,06	–	–	–
MMSE (raw score)					
All participants	33	–	24,09	5,36	9,00 – 30,00
Age group 20–59 years	12	–	27,08	2,81	21,00 – 30,00
Age group 60+ years	21	–	22,38	5,76	9,00 – 30,00
Verbal fluency (raw score)					
All participants	32	–	15,91	6,19	4,00 – 32,00
Age group 20–59 years	12	–	18,17	4,67	11,00 – 26,00
Age group 60+ years	20	–	14,55	6,69	4,00 – 32,00
Rey complex figure – copy total (raw score)					
All participants	29	–	28,03	8,34	4,00 – 36,00
Age group 20–59 years	11	–	31,86	4,28	24,00 – 36,00
Age group 60+ years	18	–	25,69	9,40	4,00 – 36,00
Rey complex figure – recall total (raw score)					
All participants	30	–	12,25	8,42	0,00 – 29,50
Age group 20–59 years	12	–	17,46	7,84	4,00 – 29,50
Age group 60+ years	18	–	8,78	7,02	0,00 – 25,50
Similarities (raw score)					
All participants	30	–	24,30	11,63	0,00 – 41,00
Age group 20–59 years	12	–	30,67	11,90	0,00 – 41,00
Age group 60+ years	18	–	20,06	9,56	0,00 – 36,00

*MMSE* Mini Mental State Examination

^a^1 minimum salary refers to BRL 788.00/month

Regarding the participants’ overall cognitive performance in the MMSE, the lowest total score was 9 points and the highest was 30 (*M* = 24.09, *SD* = 5.36) points. About, 27.3% of participants displayed substandard performance in the MMSE (8.3% in young adults group and 38.1% in older adults group).

In the Verbal Fluency test, only 9.4% of participants showed substandard performance and all deficits were presented by older adults. In the total score in the Rey Complex Figure copy section, 40% of the sample showing substandard performance (33.3% in young adults group and 44.4% in older adults group), and in the total score of the Rey Complex Figure recall section 40% of participants showing substandard performance (16.7% in young adults group and 55.6% in older adults group). In the WASI Similarities subtest, we found 73.3% of participants delivered substandard performance (50.0% in young adults group and 83.3% in older adults group).

## Discussion

Studies corroborate this finding and point out deficits in the executive performance of people with object hoarding disorder [21] and animal hoarding according a single case report [28]. Executive functions comprise several subcomponents, such as planning, logic reasoning, decision-making skills, cognitive flexibility, and inhibitory control. They allow people to control and regulate their information processing and behavior [20]. A potential explanation for this finding is that, because individuals with hoarding behaviors have these functions compromised, they would be unable to control their impulse of bringing more and more animals in and to keep an organized environment. Additionally, they would have trouble to plan according to their actual financial situation and keep a number of animals they could actually afford, in addition to ensuring the quality of their physical space.

Hartl et al. [15] found that individuals with hoarding behaviors show deficits related to their organization and planning skills when copying the Rey complex figure. The elements of the figure were drawn in a disarranged manner. Studies point out that memory deficits are also positively associated with object hoarding disorder. Hence, memory difficulties added to exaggerated negative beliefs would reinforce the hoarding behavior [13, 15]. This study also found participants had trouble copying and recalling the Rey complex figure. Therefore, we can infer that, just as individuals with hoarding behaviors show deficits in their perception, organization, planning, and visual memory skills, individuals with animal hoarding behaviors also show these same deficits. This finding may help understand the hoarding behavior in animal hoarding as it reinforces the hypothesis that organization, planning, and visual memory deficits would lead someone to lose track of the number of animals, which end up breeding in captivity. Additionally, perception deficits may lead individuals with animal hoarding behaviors to not see their animals’ actual health status or their environment’s conditions.

Cognitive decline occurs due to various biopsychosocial influences [2]. In this study, participants presented lower MMSE performance when compared to other healthy older adults and elderly [16, 17]. Reduction of the cognitive capacity can bring to the subject of damages and the tasks in daily activities [31]. We inferred that, in advanced ages, there will be a higher rate of people presenting cognitive deficits associated with animal hoarding compared to healthy people at the same ages.

Based on this study’s results, we found that people with animal hoarding show deficits related primarily to their executive functions. Out of the variables investigated, age and schooling were more closely related to the participants’ cognitive performance, as well as the number of animals accumulated. Due to differences with hoarding disorder, some researchers even propose the animals hoarding as a new disorder [9].

The findings by this study are the first of their kind given the literature lacks empiric studies measuring the cognitive performance of individuals with hoarding behaviors. As for limitations, we could point out that the sample is small and covered only the area of a city in the south of Brazil, which characterizes it as regional. In addition, it should be considered as a limitation of the study, the absence of analysis of other comorbid diagnoses. Another limitation is that it was conducted with a clinical sample yet to be investigated, the discussions and theoretical grounds were based on studies looking into people with object hoarding disorder. Thus, we suggest that further studies should be conducted, because this clinical condition remains largely unexplored by empirical studies. One more suggestion is including a healthy sample with the same demographic characteristics to make a comparison of the performance in all the test. The development of new studies will enable the development of therapeutic strategies to help the treatment and management of this problem, which translates into suffering for people, animals and their environment.

## Conclusions

Our results showed that participants have cognitive deficits related to verbal concept formation, abstract verbal reasoning, and general intellectual skills. Their perception, visuoconstructional, visual memory, planning, and organization skills and problem-solving strategies were also compromised. Therefore, we can infer they have cognitive deficits related primarily to executive functions.

## Data Availability

Data will be provided if requested.
